# From Transaction to Transformation: Building Trust in Health Systems; A Response to Recent Commentaries

**DOI:** 10.34172/ijhpm.9188

**Published:** 2025-05-26

**Authors:** Martin McKee, May CI van Schalkwyk, Rachel Greenley, Govin Permanand

**Affiliations:** ^1^Department of Health Services Research and Policy, London School of Hygiene & Tropical Medicine, London, UK.; ^2^Centre for Pesticide Suicide Prevention, University of Edinburgh, Edinburgh, UK.; ^3^Division of Country Health Policies and Systems, World Health Regional Office for Europe, Copenhagen, Denmark.

 We are grateful to Reicher,^1^ Finegood and Yakimov,^2^ Gille and colleagues,^3^ and Peters^4^ for their valuable reflections on our paper.^5^ The role of trust in health systems is, as we all agree, a complex topic. Indeed both Gille^6^ and ourselves^7^ have written books on this important topic. Consequently, their contributions, which examine additional concepts and proposed actions, are very welcome.

 Reicher emphasizes the importance of building trust between the public and authorities, particularly in the context of public health initiatives.^1^ He argues that trust is rooted in shared identity, where both the public and authorities see themselves as part of a single, unified group. He proposes four practical guidelines for building trust: (1) trust the people, recognising their resilience and capabilities; (2) respect differences, acknowledging the diverse circumstances and barriers faced by different groups; (3) engage with the public, involving them in decision-making processes and working through trusted community leaders; and (4) prioritise understanding and support over blame and punishment, addressing non-adherence to political and public health measures with empathy and practical assistance. He highlights lessons from the COVID-19 pandemic, noting that trust in governmental and health authorities was crucial for vaccine uptake and adherence to public health measures. We agree and, elsewhere, we have described international experiences in co-creation of responses to the pandemic.^8^ Reicher underscores how effective trust-building requires sincere and meaningful actions, not just rhetorical commitments, and calls for a paradigm shift in how authorities interact with the public, moving from a transactional to a relational approach. This approach underpinned the creation of Independent SAGE, a group of scientists in the United Kingdom that included Reicher and one of us (MM) who believe openness and transparency leads to better understanding and better decision-making. This group came together during the pandemic with the goal of engaging in conversations with a public that was often confused and fearful.^8^ As this implies, we also agree with him that building trust is essential for effective crisis management and public health, advocating for interdisciplinary collaboration and continuous engagement with the public to foster a sense of shared identity and mutual trust.

 Finegood and Yakimov remind us of the need for a systems-thinking approach when dealing with complexity.^2^ We agree and have frequently employed systems thinking to understand how health systems function. Recent examples include our analysis of medical schools in the United Kingdom^9^ and cancer screening.^10^ When working with human systems, success requires the alignment of a series of sub-systems, each interacting in ways that are complex, by which we mean subject to path dependency and characterised by feedback loops and non-linear relationships.^11^ We agree with their view that authentic trust, which is built through mature, articulated, and carefully considered relationships, is essential. We also agree that characteristics of some health systems, such as command-and-control structures and cultures of fear and risk avoidance, are to be avoided as this can lead to “cordial hypocrisy” or pretend trust. Again, this is something that we have written about extensively elsewhere, for example, in our work on the role of targets in healthcare.^12^ There we argued that these could have a positive role where they were co-created in an inclusive process with stakeholders but, where they were imposed top down, they often led to gaming, opportunism, and in some cases deceit. We thus also agree with them that efforts should be directed towards building reciprocal relationships and fostering environments that support collaboration, learning, and adaptability. So too that principles, rather than rigid rules, should guide trust-building efforts, and that trust should be viewed as an ongoing process.

 We fully endorse the emphasis by Gille and colleagues on the critical role of public trust in the effectiveness and legitimacy of health systems. They argue that trust should be deeply integrated into health policy-making, from design to implementation, assessment, and evaluation.^3^ We especially appreciate their proposals for building public trust: understanding trust before attempting to build it, providing tailored guidance on trust-building, developing trust performance indicators, and implementing targeted communication strategies. We too have emphasised the complexity of trust,^7^ noting that it is context-specific and influenced by cultural norms, early experiences, and societal values. We concur with their stress on the importance of proactive trust-building measures, such as continuous and deliberate interventions, and the need for clear leadership and commitment. Their insights into the challenges of measuring trust are especially welcome, as this is something that we have wrestled with, discussing the challenges at length in our book.^7^ We endorse their call for dynamic and holistic approaches that go beyond conventional survey designs. We particularly welcome their proposal for the use of Trust Performance Indicators that would routinely collect data on trustworthiness, which can inform policy processes and health system interventions. This is something that we have called for, although disappointingly, perhaps unsurprisingly, we have found little support for it among policy-makers.

 Peters addresses the broader crisis of trust in public institutions and its implications for health systems.^4^ This is something that we have also addressed elsewhere, in particular in our work on the association between poor health and the growth of populism.^13^ We fully agree with him that trust in health systems is intertwined with trust in other public institutions and societal trust more broadly. We support the actions that he identifies as ways to build trust in public institutions, especially at a time when these institutions are coming under unprecedented attack in the United States and elsewhere.^14^ These are strengthening the rule of law, enhancing civic participation, promoting government transparency, and demonstrating ethical leadership. He also highlights the role of health systems in fostering economic inclusivity and social protection, and improving government responsiveness and service delivery, again something that we have argued for elsewhere.^4^ We found his focus on trustworthiness helpful, emphasising the importance of credibility, participation, reliability, ethical behaviour, and transparent communication. As we have written elsewhere: “Trustworthiness is a key characteristic of a well-performing health system, built up over time, but it should not be assumed. Sometimes mistrust of the health system is well placed and helps to expose systems that are unsafe, prejudiced and lacking in appropriate governance and resources.”^7^

 Once again, we thank the authors of all these papers and appreciate their contribution to extending the points we raise in our commentary. Thanks to their engagement with our initial paper, this collection of commentaries represents an excellent starting point for those seeking to understand the critical role of trust in health systems and in sustaining a healthy, safe, and inclusive society.

## Acknowledgement

 This response draws on work undertaken by the authors in the course of their work for the World Health Organization (WHO) Europe or the European Observatory on Health Systems and Policies but no specific funding was required for writing it.

## Ethical issues

 Not applicable.

## Conflicts of interest

 The authors are either employees of WHO (GP) or received support from WHO and the Observatory to attend and participate in the WHO conference on which this work is based.
